# A short-term clinical evaluation of immediate implant placement in periodontitis patients

**DOI:** 10.3389/fcimb.2025.1555964

**Published:** 2025-04-01

**Authors:** Guojiang Li, Xufeng Chen, Yujia Feng, Ying Lu, Wanlin Liao, Rui Huang, Ejiao Yang, Renfa Lai, Zhiqiang Feng

**Affiliations:** ^1^ Hospital of Stomatology, the First Affiliated Hospital of Jinan University, Guangzhou, China; ^2^ School of Stomatology, Jinan University, Guangzhou, China; ^3^ Department of Stomatology, Nanfang Hospital, Southern Medical University, Guangzhou, China; ^4^ Guangdong Second Provincial General Hospital (Guangdong Provincial Emergency Hospital), Guangzhou, China

**Keywords:** periodontitis, immediate implant placement, implant survival, marginal bone loss, peri-implant health, retrospective analysis, Kaplan-Meier survival analysis

## Abstract

**Background:**

With the advancement of oral implant technology, immediate implant placement is believed to be feasible for periodontitis patients. However, there is a lack of high-quality clinical studies regarding this approach. This study aimed to observe the short-term implant survival rate and conditions of peri-implant tissues in periodontitis patients who received immediate implants without systematic periodontal treatment.

**Methods:**

This retrospective study included 95 patients and 234 implants treated at the Stomatological Hospital of Jinan University from June 2017 to December 2022. Patients were classified according to the 2018 AAP/EFP periodontal classification system, with Stage determined by CBCT-assessed marginal bone loss (MBL) and Grade estimated based on annual bone loss rate, smoking status, and diabetes history. Immediate implant placement was performed following atraumatic tooth extraction, with bone defects augmented using Bio-Oss bone graft and covered with Bio-Gide collagen membrane as needed. Patients were followed up for 12 months, during which implant survival, modified sulcus bleeding index (mSBI), modified plaque index (mPLI), marginal bone loss (MBL), and peri-implant probing depth (PPD) were assessed.

**Results:**

A total of 95 patients (234 implants) were included, with a mean age of 58.59 years. The distribution of Stage II-IV and Grade A-C periodontitis was recorded. Preoperative assessments showed a significant increase in P-PDD, CAL, and MBL with greater disease severity (p < 0.001). One-year follow-up data indicated an implant survival rate of 97.86%, with Kaplan-Meier survival analysis revealing significantly lower survival rates in Stage IV and Grade C patients (p < 0.05). Postoperative soft tissue health assessment showed significant differences in mSBI and mPLI between stages (p = 0.002, p = 0.007) but not grades (p > 0.05). PPD did not differ significantly among groups (p > 0.05), whereas MBL was significantly higher in Stage IV than in Stage II and III (p < 0.001), though no significant differences were observed across grades (p > 0.05). Clinical and radiographic evaluations demonstrated favorable implant outcomes, with most patients reporting high satisfaction. These findings reinforce the viability of immediate implant placement in periodontitis patients, demonstrating high short-term success rates across different disease severities. While disease severity and progression rate may influence clinical outcomes, appropriate case selection, meticulous surgical techniques, and comprehensive postoperative care can lead to predictable and favorable implant success, even in patients with periodontitis.

## Background

With the continuous advancement of oral implant technology, clinical research confirms the feasibility of immediate implants for periodontitis patients. Periodontitis is a prevalent periodontal disease seen in clinical practice (Arboleda et al., 2019). As the name suggests, it is a chronic inflammation of the tissue surrounding the tooth, which leads to the gradual loss of attachment. Typically, the inflammation progresses towards the root of the tooth, eventually causing the tooth to fall out due to the gradual absorption of the alveolar bone. Alveolar bone resorption is the primary clinical effect of periodontitis, and it is the primary reason for dental defects or tooth loss. Because periodontitis causes damage to soft tissue and destruction of alveolar bone, it has been considered a contraindication for immediate implantation.

Dental implantation is a common treatment for dental defects or tooth loss. Dental implant surgeries are classified by placement method into submerged and non-submerged types. Additionally, the methods of dental implantation can be divided into traditional delayed implant surgery, early implant surgery and immediate implant surgery (Panchal et al., 2022). Delayed implantation is usually performed within six months after tooth loss, while immediate implantation involves placing dental implants immediately after tooth extraction, without waiting for the extraction site to heal (dos Santos Canellas et al., 2019; Woods et al., 2019). Immediate implantation has several advantages and has been widely promoted and applied in clinical settings. Such as, it decreases the overall treatment duration and costs, minimizes the need for multiple surgeries, helps in maintaining the integrity of the alveolar ridge, enhances patient comfort and satisfaction, and improves osseointegration due to the healing properties of a freshly extracted socket (Ketabi et al., 2016; Mustakim et al., 2023). It has achieved high success rates. The first literature on immediate implantation dates back to 1978, when Schulte published a paper on the topic (Schulte et al., 1978). In the early 1990s, Lazzara reintroduced the concept of immediate implantation (Del Fabbro et al., 2009). Several years later, Geb identified immediate implantation as a satisfactory treatment in the literature, with 50 patient follow-ups indicating successful outcomes. Clinically, immediate implantation methods can reduce pain and the frequency of dental visits, which can result in insufficient bone mass in the implantation area. Immediate implants are also beneficial in achieving ideal implant positioning for maintaining the natural shape of the soft tissue of the gums, resulting in desirable aesthetic effects (Erkapers et al., 2017; Ragucci et al., 2020). In 2013, Gustavo Cabello and his team utilized a tri-modal approach for aesthetic zone implantation, which included immediate placement after tooth extraction, flapless procedure, and immediate provisional restoration. They evaluated the changes in surrounding soft tissues and their correlation with gingival and periodontal biotypes. The study concluded that immediate implantation, combined with flapless surgery and immediate provisional restoration, leads to superior aesthetic results and reduced complications (Cabello et al., 2013).

With the ongoing advancement of oral implant technology, clinical studies have indicated that immediate implants are a feasible option for periodontitis patients, albeit with a higher risk. This also implies that immediate implant surgery demands higher requirements in terms of the operator’s skill, the selection of surgical cases, and the rationality of the surgical plan. Moreover, if clinical surgical studies can overcome some of the limitations of immediate implantation in the alveolar fossa due to periodontal infection, desirable outcomes can be achieved (Crespi et al., 2010; Villa et al., 2010; Alghandour et al., 2018; Bakkali et al., 2021). Novaes and colleagues found that intraoperative debridement, postoperative antibiotics, and postoperative attention to oral hygiene can achieve satisfactory results in the immediate treatment of untreated periodontitis teeth (Novaes and Novaes, 1995; Velasco-Ortega et al., 2018). In a systematic review by Reda, it was suggested that the use of extraoral bonding can reduce the residue of adhesives and decrease the occurrence of peri-implant lesions. Additionally, the use of eugenol-free oxide cement results in no residue in soft tissues (Reda et al., 2022). This study focused on peri-implant tissues in periodontitis patients who received immediate implants without prior periodontal treatment. Initially, it analyzed the clinical indicators of implants in patients across various stages of periodontitis, comparing these with extensive patient clinical data. Subsequently, the research involved a detailed comparison and analysis of both soft and hard tissues surrounding the implant. This comprehensive approach was undertaken to provide a robust reference for the implementation of immediate dental cavity implantation specifically in periodontitis patients.

## Methods and materials

### Study design and participants

This retrospective study was conducted at the Stomatological Hospital of Jinan University, Guangzhou, China, from June 2017 to December 2022. A total of 95 cases with 234 implants were included. Patient demographic data, smoking history, and diabetes status were recorded before implant placement. All implants were placed at sites where teeth were extracted due to periodontitis, and all patients met the clinical diagnostic criteria for periodontitis.

### Preoperative data collection

To assess the periodontal condition of the extracted teeth before immediate implant placement, each tooth was classified according to the 2018 American Academy of Periodontology (AAP) and European Federation of Periodontology (EFP) classification system (**Table 1**). The Stage (severity of periodontitis) was based on marginal bone loss (MBL, % of root length), assessed through CBCT imaging and periodontal probing before extraction. The Grade (progression rate) was estimated based on annual bone loss relative to patient age and adjusted according to smoking status and diabetes history (Caton et al., 2018; Tonetti et al., 2018a).

**Table 1:** Criteria for stage and grade assignment.

	Bone Loss (MBL as % of Root Length)	Clinical Attachment Loss (CAL, mm)	Complexity of Defects
Stage I	<15%	1-2mm	No major defects
Stage II	15-33%	3-4mm	Mild bone loss
Stage III	>33%	≥5mm	Possible furcation involvement
Stage IV	>50%	≥5mm	Severe structural loss

Grade (Progression Rate)	Bone Loss/Age (%/years)	Smoking (cigarettes/day)	Diabetes
Grade A	<0.25	Non-smoker	No
Grade B	0.25-1.0	<10	Possible
Grade C	>1.0	≥10	Yes

Clinical attachment loss (CAL) was measured using a Florida periodontal probe with a standardized 0.25 N probing force. Measurements were taken at six sites per tooth—mesiobuccal, mid-buccal, distobuccal, mesiolingual, mid-lingual, and distolingual. CAL was calculated as:


CAL = PPD + (CEJ−GM)

where CEJ-GM was recorded as negative in cases of gingival overgrowth and positive in cases of gingival recession.

Preoperative marginal bone loss (MBL) was assessed using cone-beam computed tomography (CBCT) to evaluate alveolar bone levels. MBL was defined as the vertical distance from the cementoenamel junction (CEJ) to the alveolar crest (AC), measured at the mesial (M) and distal (D) aspects of each tooth. Bone loss rate was calculated as:


Bone Loss Rate =(MBL/Root Length)× 100%

CBCT images were analyzed by a single experienced examiner using standardized imaging protocols to minimize variability.

Each extracted tooth was assigned a Stage and Grade before immediate implant placement, and the impact of different periodontal conditions on the survival and peri-implant tissue response was analyzed accordingly.

Surgeries were performed in a sterile implanted operating room by one experienced implant surgeon. This study was reviewed and approved by the Institutional Review Board (IRB) of the First Affiliated Hospital of Jinan University, with the approval number: KYK-2022-020. All participants/patients provided informed consent to participate in the study. All participants/patients provided informed consent for the publication of their anonymized case details and images.

### Inclusion criteria

1) Extracted teeth were diagnosed with chronic periodontitis and classified according to the 2018 AAP/EFP periodontal classification system, with Stage and Grade determined before extraction based on clinical attachment loss (CAL), marginal bone loss (MBL), smoking history, and systemic conditions; 2) Patients had no uncontrolled systemic diseases that could contraindicate implant surgery, including: No uncontrolled diabetes (blood glucose < 8 mmol/L). No coronary heart disease or malignant tumors. No osteoporosis or other systemic conditions that would contraindicate oral surgical procedures. All patients were deemed fit for surgery based on their medical history and clinical evaluation (Gómez-de-Diego et al., 2014); 3) All affected teeth were assessed by an experienced periodontist, confirmed as having no preservation value, and indicated for extraction due to advanced periodontal destruction (Stage II-IV); 4) Pre-extraction CBCT confirmed sufficient bone mass for immediate implantation: Minimum residual alveolar bone height of ≥4 mm after extraction (excluding the apex). Adequate buccal and lingual bone walls to support primary stability (Yalcin et al., 2009); 5) Patient demonstrated good compliance, maintained satisfactory post-operative oral hygiene, and signed informed consent; 6) Patients were aged 18-92 years with sufficient physical health to undergo surgery and follow-up examinations.

### Exclusion criteria

1) Severe anatomical limitations that could interfere with implant placement, including: Insufficient residual bone volume after extraction, confirmed by CBCT. Critical anatomical structures (e.g., maxillary sinus, inferior alveolar nerve) interfering with implant positioning. Severe malocclusion, severe skeletal discrepancies, or extensive occlusal dysfunction that could compromise implant function.; 2) Patients with parafunctional habits that could negatively impact implant survival, including: Severe bruxism or clenching habits that could not be effectively managed with occlusal therapy; 3) Patients with systemic conditions contraindicating implant surgery, including: Long-term use of bisphosphonates (risk of osteonecrosis). Uncontrolled metabolic diseases, such as uncontrolled diabetes (blood glucose >8 mmol/L). Severe osteoporosis or other systemic disorders affecting bone metabolism; 4) Patients with high-risk lifestyle factors, including: Heavy smoking (≥20 cigarettes/day). Alcohol dependence or substance abuse; 5) Patients with psychiatric disorders or cognitive impairments that could affect post-operative compliance and follow-up; 6) Cases with incomplete clinical records or missing pre-extraction periodontal data, preventing accurate classification of Stage and Grade.

### Surgical procedure and materials

All surgeries were performed in a sterile operating room by a single experienced implant surgeon. After local anesthesia, teeth were atraumatically extracted using a minimally invasive extraction device (Original Luxator, Direta, Sweden). Granulation and inflammatory tissue were debrided, and extraction sockets were irrigated with saline and 3% hydrogen peroxide. Implants (Bioconcept, China) were placed with a torque of ≥35 N.cm, and the implant platform was positioned 1.5-2 mm below the alveolar crest. Bone defects were augmented with Bio-Oss bone graft (Geistlich, Switzerland) and covered with Bio-Gide collagen membrane as needed. Postoperative CBCT was taken to confirm implant positioning, and patients underwent a standardized post-surgical care protocol, including anti-infective therapy for three days and suture removal at two weeks.

Patients were followed up at 12 months postoperatively to evaluate implant survival, modified sulcus bleeding index (mSBI), modified plaque index (mPLI), peri-implant probing pocket depth (PPD), and marginal bone loss (MBL). Standardized clinical measurements were performed by the same experienced examiner.

Although this study is retrospective in nature, it has a prospective component, as all included cases were followed up within a standardized 12-month period after case collection, ensuring consistency in data collection and clinical evaluation.

The materials used in the implant surgery were surface tomography (Sirona, USA), CBCT and supporting software (Setke, France, NNT), dental implanter (Bian, Switzerland), bone level implant(Bioconcept, China),Bio-Oss bone powder (Geistlich, Switzerland), Bio-Gide absorbable biofilm (Geistlich, Switzerland), minimally invasive extraction device (Original Luxator, Direta, Sweden), and periodontal probe (Florida).

### Evaluation of tooth prognosis

To evaluate the prognosis of the implants, we observed the implant survival rate and changes in the surrounding soft and hard tissues for 12 months after immediate implant placement. The implant survival rate was assessed based on the criteria set by Alberksson and Zarb (1986) (Kuchler et al., 2016). Marginal bone loss (MBL) around the implant was evaluated using CBCT at baseline and 12 months postoperatively, measuring the distance from the implant platform to the most crown-bone contact point.

Additionally, we recorded the modified sulcus bleeding index (mSBI), modified plaque index (mPLI), and peri-implant probing pocket depth (PPD). The clinical diagnostic criteria for peri-implantitis were used to assess these indices. For PPD, measurements were taken at six predefined sites (mesiobuccal, mid-buccal, distobuccal, mesiolingual, mid-lingual, and distolingual) using a standardized Florida periodontal probe with a 0.25 N probing force. The mean PPD value for each implant was used for analysis.

### Statistical analysis

Statistical analysis was performed using SPSS version 22.0. Kaplan-Meier survival analysis was used to evaluate implant survival, with Log-Rank (Mantel-Cox) test for between-group comparisons. One-way ANOVA was applied to compare PPD and MBL across groups, using Bonferroni’s test or Tamhane’s T2 test for *post hoc* analysis. Kruskal-Wallis H test was used for ordinal variables (mSBI, mPLI), followed by Mann-Whitney U test for pairwise comparisons. Statistical significance was set at p < 0.05, with Bonferroni correction for multiple comparisons.

## Results

### Patient characteristics, severity of periodontitis, and preoperative condition

As shown in **Table 2**, a total of 95 patients (59 males and 36 females) with 234 implants were included in this study. The patients had an age range of 18 to 92 years, with a mean age of 58.59 years. Among the patients, 50 (52.63%) were aged ≥60 years, while 45 (47.37%) were aged <60 years. For the implants, 134 (57.26%) were placed in patients aged ≥60 years and 100 (42.74%) in patients aged <60 years. Smoking status revealed that 48 patients (50.53%) were non-smokers, 29 (30.53%) were moderate smokers, and 18 (18.94%) were heavy smokers. The implants were similarly distributed, with 111 (47.44%) placed in non-smokers, 69 (29.49%) in moderate smokers, and 54 (18.94%) in heavy smokers. Regarding diabetes status, 71 (74.74%) patients were non-diabetic, 18 (18.95%) were at risk for diabetes, and 6 (6.32%) were diabetic. For implants, 165 (70.51%) were placed in non-diabetic patients, 58 (24.79%) in patients at risk for diabetes, and 11 (4.70%) in diabetic patients. In terms of periodontitis stage, no patients or implants were classified as Stage I. Stage II was observed in 27 (28.42%) patients and 61 (26.07%) implants, Stage III in 36 (37.89%) patients and 110 (47.01%) implants, and Stage IV in 32 (33.68%) patients and 63 (26.92%) implants. For periodontitis grade, Grade A was seen in 44 (46.32%) patients and 82 (35.04%) implants, Grade B in 33 (34.74%) patients and 93 (39.74%) implants, and Grade C in 18 (18.95%) patients and 59 (25.21%) implants.

**Table 2:** The basic characteristics of cases [n (%)].

	Patients (n=95)	Implants (n=234)
Gender		
Male	59 (62.11)	161 (68.80)
Female	36 (37.89)	73 (31.20)
Age		
≥60 years	50 (52.63)	134 (57.26)
<60 years	45 (47.37)	100 (42.74)
Smoke		
Non-smokers	48 (50.53)	111 (47.44)
Moderate smokers	29 (30.53)	69 (29.49)
Heavy smokers	18 (18.94)	54 (18.94)
Diabetes		
Non-diabetic	71 (74.74)	165 (70.51)
At risk for diabetes	18 (18.95)	58 (24.79)
Diabetic	6 (6.32)	11 (4.70)
Stage		
I	0 (0.00)	0 (0.00)
II	27 (28.42)	61 (26.07)
III	36 (37.89)	110 (47.01)
IV	32 (33.68)	63 (26.92)
Grade		
A	44 (46.32)	82 (35.04)
B	33 (34.74)	93 (39.74)
C	18 (18.95)	59 (25.21)

**Figure 1:**
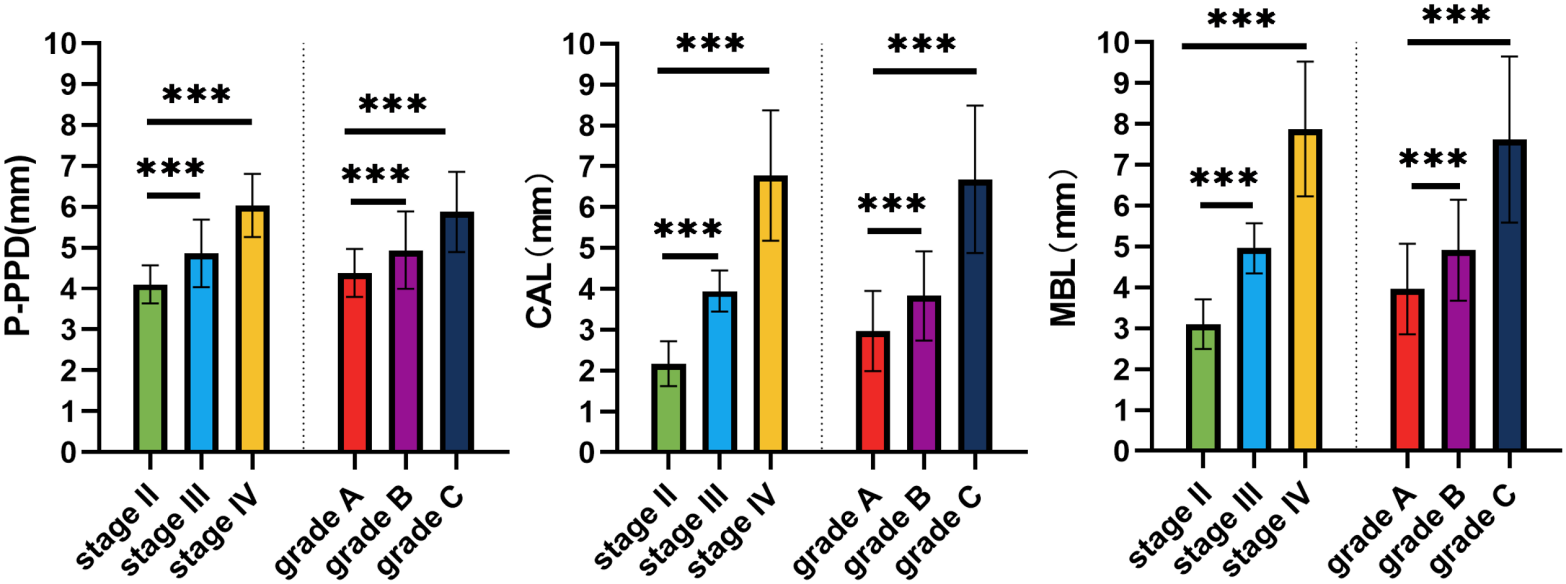
Comparison of preoperative probing pocket depth (P-PDD), clinical attachment loss (CAL), and marginal bone loss (MBL) across different stages and grades of periodontitis. The data show that Stage IV and Grade C exhibit significantly higher values in PPD, CAL, and MBL compared to Stage II and Stage III, and Grade A and Grade B (***,*p*<0.001). Error bars represent standard error of the mean. *** indicates statistically significant difference at p < 0.001.

**Table 3** and **Figure 1** present the initial periodontal condition of patients classified by stage and grade. Preoperative probing pocket depth (P-PDD), clinical attachment loss (CAL), and marginal bone loss (MBL) were measured in patients at the time of initial diagnosis. The results indicate that the severity of periodontal disease increased (from Stage II to IV, and Grade A to C), P-PDD, CAL, and MBL values all significantly increasing. One-way ANOVA was performed to assess the differences between stages and grades, and the results indicated that these differences were statistically significant (p < 0.001). Stage IV patients exhibited the highest mean P-PDD (6.03 ± 0.76 mm), followed by Stage III (4.86 ± 0.82 mm) and Stage II (4.10 ± 0.47 mm). Similarly, Grade C patients had the highest P-PDD (5.90 ± 0.96 mm), while Grade A patients had the lowest (4.39 ± 0.59 mm). CAL measurements showed a similar trend. Stage IV patients had the highest mean CAL (6.76 ± 1.56 mm), while Stage II patients had the lowest (2.17 ± 0.55 mm). Grade C patients had significantly higher CAL than Grade A and B, with a mean of 6.67 ± 1.76 mm compared to 2.97 ± 0.98 mm for Grade A. Marginal bone loss was most prominent in Stage IV (7.81 ± 1.64 mm), followed by Stage III (4.96 ± 0.61 mm) and Stage II (3.10 ± 0.60 mm). Likewise, Grade C had the highest MBL (7.56 ± 2.00 mm), compared to Grade A (3.81 ± 1.23 mm).

**Table 3:** The condition of periodontal soft tissue at the time of initial diagnosis (x ± s)/mm.

	Stage II	Stage III	Stage IV	Grade A	Grade B	Grade C
P-PPD	4.10 ± 0.47	4.86 ± 0.82	6.03 ± 0.76	4.39 ± 0.59	4.94 ± 0.95	5.90 ± 0.96
CAL	2.17 ± 0.55	3.95 ± 0.50	6.76 ± 1.56	2.97 ± 0.98	3.83 ± 1.09	6.67 ± 1.76
MBL	3.10 ± 0.60	4.96 ± 0.61	7.81 ± 1.64	3.96 ± 1.11	4.91 ± 1.23	7.56 ± 2.00

### The implant retention rate after 1 year of surgery

**Figure 2:**
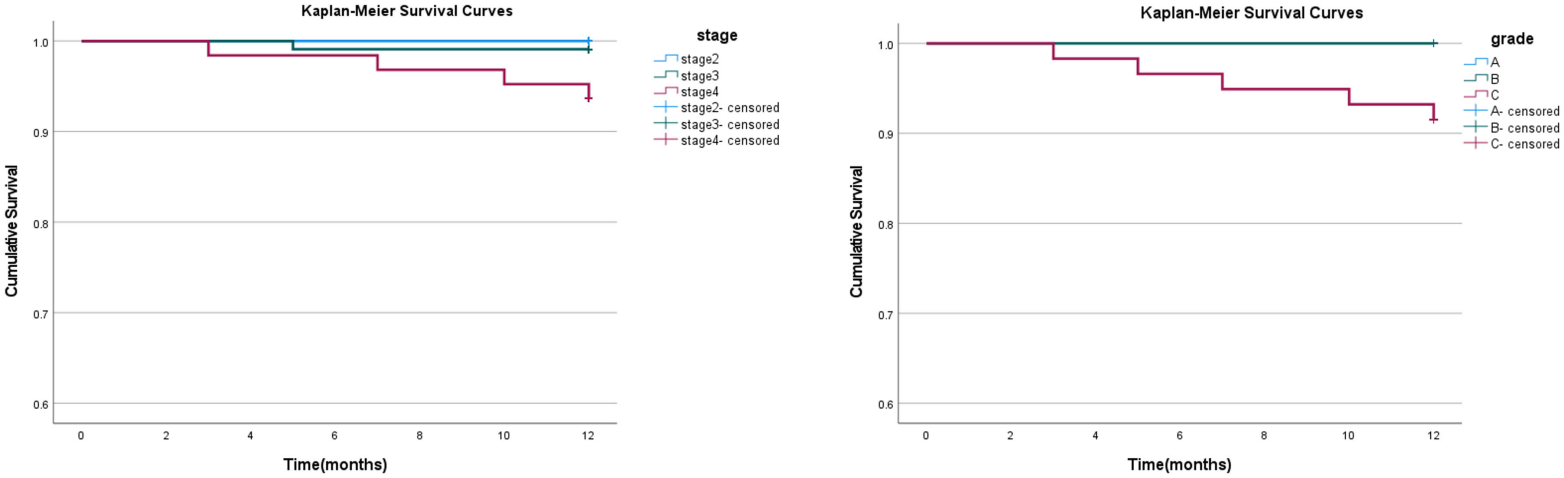
Kaplan-Meier survival curves for implants grouped by stage and grade. The left panel shows the survival curves for different stages of periodontitis (stage II, III, IV), and the right panel shows the survival curves for different grades (A, B, C). The curves for Grade A and Grade B overlap, indicating similar survival rates, while the curve for Grade C shows a distinct pattern. Censoring is indicated by the marks on the curves.

The implant survival rate is a key indicator of the success of implant surgery. In this study, the 1-year survival rate of 97.86% was derived from Kaplan-Meier (K-M) survival analysis, based on 234 implants (see **Table 4**, **Figure 2**). This analysis accounts for the time-to-event data and censored observations. A total of 5 implants failed during the 12-month follow-up, with failure events occurring at 3, 5, 7, 10, and 12 months. Specifically, 3 implants became loose after the implant was placed. Although they initially achieved adequate primary stability, they failed to maintain proper osseointegration during subsequent healing, leading to implant mobility. X-ray examination revealed insufficient bone density surrounding these implants. One implant experienced a localized infection, characterized by soft tissue redness and mild discharge. Despite treatment, it failed to fully heal and was eventually lost, being classified as a failure. Finally, one implant was exposed, likely due to inadequate soft tissue coverage, resulting in the loss of function and classification as a failure.

**Table 4:** Survival rate of implants with varying degree of periodontitis (n).

	Stage II	Stage III	Stage IV	Grade A	Grade B	Grade C
Survive	61	109	59	83	92	54
Failure	0	1	4	0	0	5
Total	61	110	63	83	92	59

For stage-based grouping, the survival rates were 100% for stage II, 99.09% for stage III, and 93.65% for stage IV. The Log-Rank (Mantel-Cox) test revealed significant differences in survival curves across stages (χ² = 7.477, p = 0.024). *Post-hoc* pairwise comparisons showed no significant difference between stage II and stage III (p = 0.456), while significant differences were observed between stage II and stage IV (p = 0.046), as well as between stage III and stage IV (p = 0.041).

In contrast, when grouped by grade, the survival rates were 100% for grade A and B, and 91.5% for grade C. The Log-Rank (Mantel-Cox) test revealed a significant difference in survival curves between the grade groups (χ² = 15.355, p < 0.001). Significant differences were found when comparing grade C with both grade A (p = 0.007) and grade B (p = 0.004), indicating that grade C exhibited lower survival rates than grade A and grade B.

### Postoperative implants condition of soft and hard tissue

**Figure 3:**
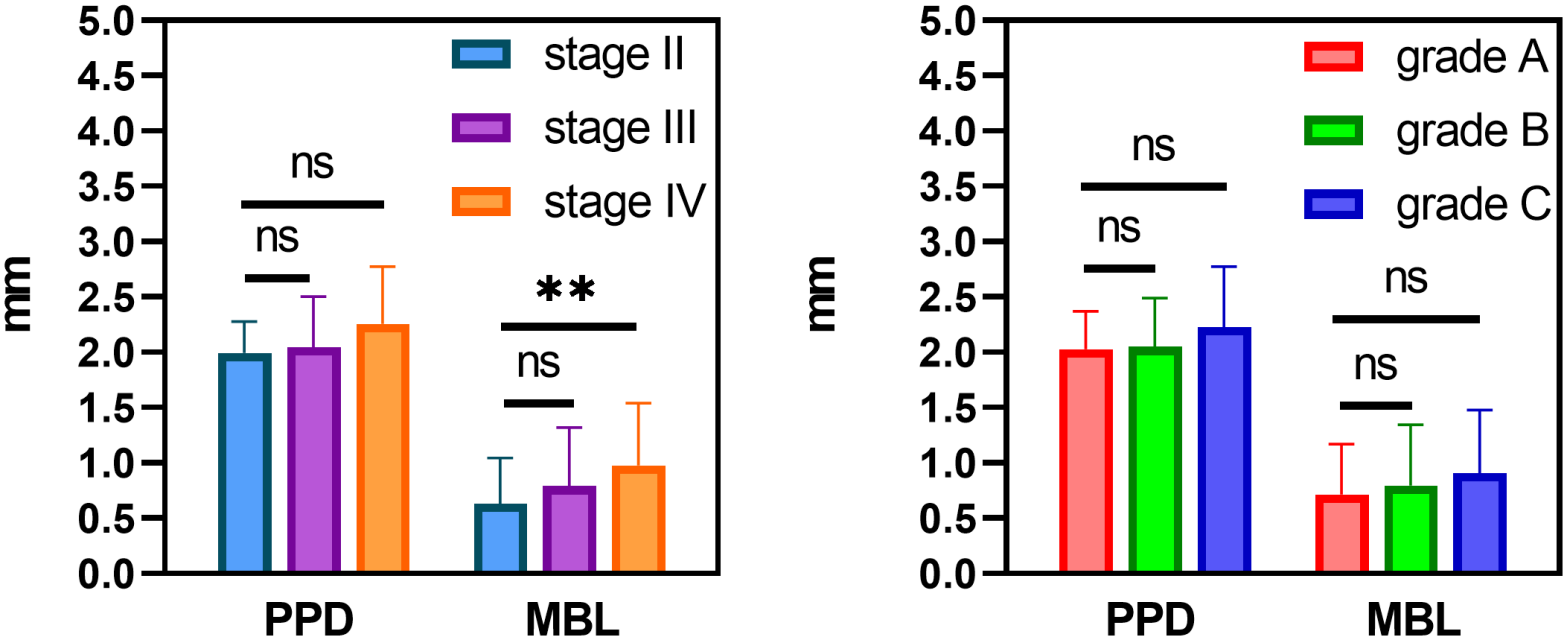
The results of PPD and MBL with different stage and grade periodontitis after 1-year immediate implant surgery. Significant differences in both PPD and MBL were observed across stages and grades. Statistical significance is indicated by n.s. (p > 0.05), and ** (p < 0.001).

Out of the 234 implants initially included in the study, 5 implants failed during the follow-up period. The final analysis was conducted using data from 229 successful implants. Therefore, the tissue around the implants was analyzed in our study (see **Table 5**, **Figure 3**). We measured the soft and hard tissue condition around the implants by observing mSBI, mPLI, MBL, and PPD at 12 months after immediate implant placement. The **Table 4** presents the mSBI, mPLI, PPD, and MBL measurements in various groups, including different stages (Stage II, Stage III, Stage IV) and grades (Grade A, Grade B, Grade C) of periodontitis. mSBI and mPLI data are reported as frequencies across different categories (0, 1, 2, 3) within each group. Kruskal-Wallis H (K-W H) test was used to assess the differences between groups, with significant differences found for mSBI across stages (p = 0.002) and mPLI across stages (p = 0.007). There were no significant differences observed in mSBI and mPLI across grades (p = 0.075 and p = 0.051, respectively). Therefore, PPD and MBL data are reported as mean ± standard deviation. One-way ANOVA was used to analyze the differences between groups, with no significant differences in PPD across stages (p = 0.120) and grades (p = 0.425). However, significant differences were observed in MBL across stages (p = 0.001) but no significant differences across grades (p = 0.115). The results of PPD and MBL in different stages (Stage II, Stage III, and Stage IV) and grades (Grade A, Grade B, and Grade C) of periodontitis after 1-year immediate implant surgery are presented in **Figure 2**. For PPD, no significant differences were observed across the stages (p > 0.05), as indicated by the ns symbol. Similarly, there were no significant differences in PPD across the grades (p > 0.05). However, for MBL, significant differences were observed between Stage IV and the other stages (Stage II and Stage III), with Stage IV showing a higher mean value (p < 0.001). No significant differences were found in MBL across grades (p > 0.05).

**Table 5:** mSBI, mPLI, PPD and MBL of implants in various groups(n=229).

Group	mSBI				mPLI			
	0	1	2	3	0	1	2	3
Stage II	3	40	17	1	7	44	10	0
Stage III	11	47	42	9	17	67	20	5
Stage IV	1	22	29	7	1	38	12	8
K-W H	12.112				9.921			
P	0.002				0.007			

Group	mSBI				mPLI			
	0	1	2	3	0	1	2	3
Grade A	3	49	25	5	9	55	17	1
Grade B	9	38	38	4	15	54	16	4
Grade C	3	22	25	8	1	40	9	8
K-W H	5.185				5.954			
P	0.075				0.051			

Group	PPD				MBL			
Stage II	1.99 ± 0.28				0.63 ± 0.42			
Stage III	2.30 ± 1.39				0.79 ± 0.53			
Stage IV	2.37 ± 1.01				0.97 + 0.56			
P	0.120				0.001			

Group	PPD				MBL			
Grade A	2.11 ± 0.84				0.71 ± 0.46			
Grade B	2.28 ± 1.33				0.79 ± 0.55			
Grade C	2.34 ± 1.04				0.91 + 0.57			
P	0.425				0.115			

**Figure 4:**
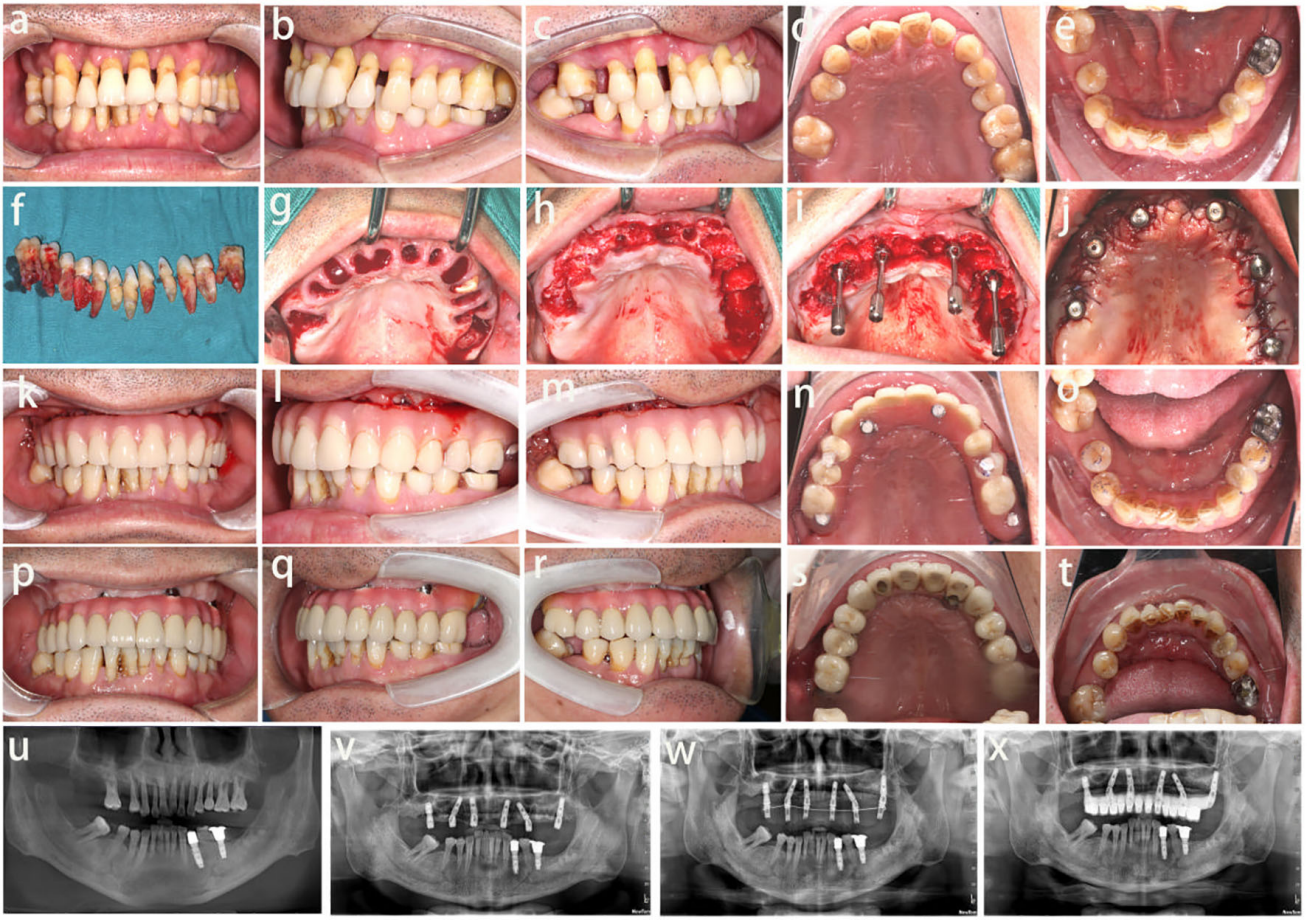
Photographic and x-ray evaluation of the oral condition. **(a-e)** The oral condition before surgery, **(f-j)** The oral condition during surgery, **(k-o)** The oral condition after surgery immediately, **(p-t)** The oral condition at 6 months after surgery, **(u-x)** The x-ray results, before immediate implant placement **(u)**, during the surgery **(v)**, after surgery immediately **(w)** and 6 months after the surgery **(x)**.

**Figure 5:**
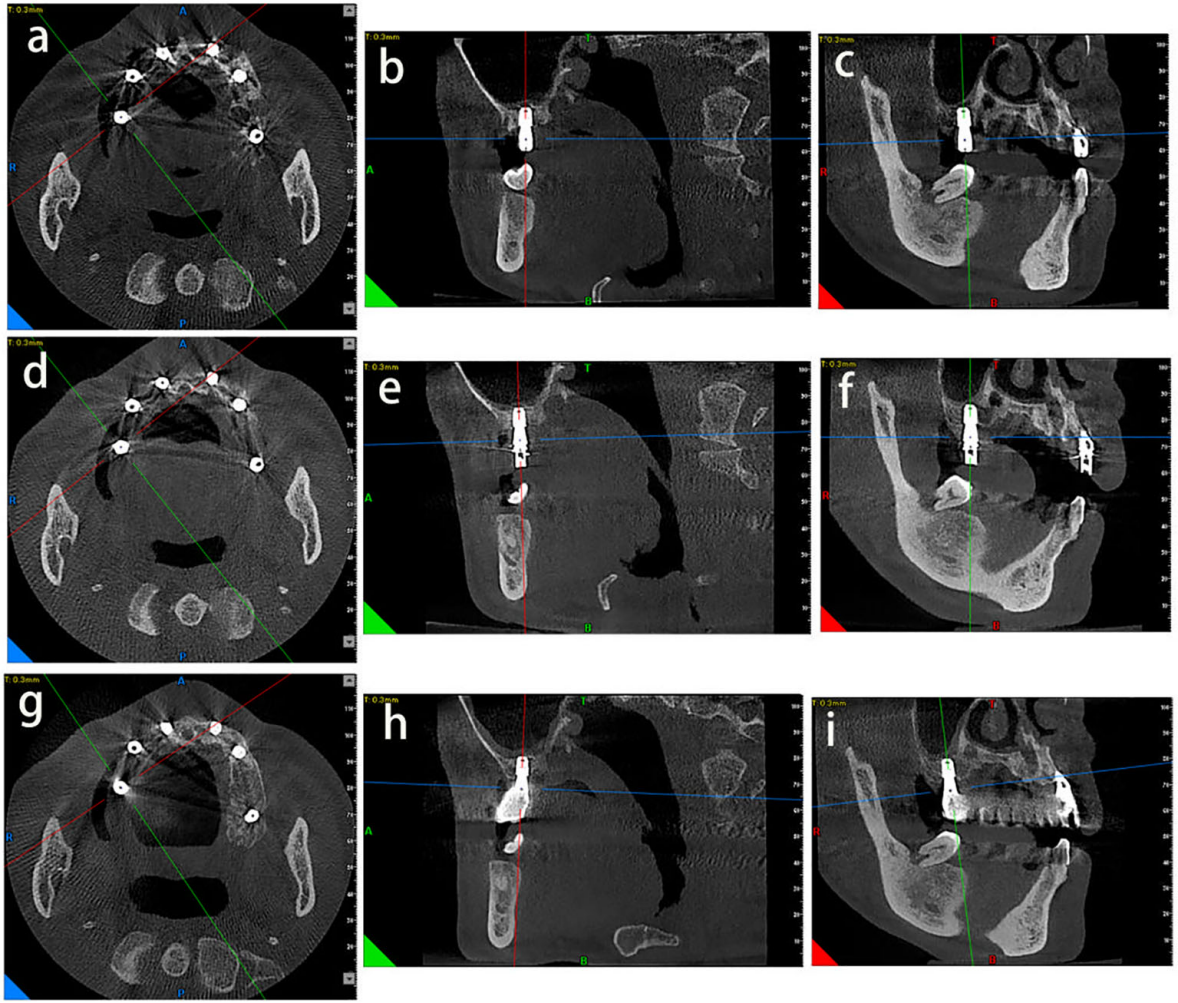
The measurement results at different time points after immediate implant placement. **(a-c)** Measurement results were measured after immediate implant placement at axial, sagittal and coronal CBCT scans immediately. **(d-f)** Measurement results were measured at 3months after immediate implant placement at axial, sagittal and coronal CBCT scans. **(g-i)** Measurement results were measured at 6 months after immediate implant placement at axial, sagittal and coronal CBCT scans. The tooth bit is 16 in this patient.

The above results showed that most of the patients are satisfied with the immediate implant placement. One of typical patient cases is a male, 52 years old, with periodontitis stage IV and grade B (**Figures 4a–e, u**). And the patient has good clinical and radiation results after immediate implant surgery (**Figures 4f–t, v–x**). Meanwhile, many measurement parameters were checked in this patient, for example, MBL were measured by CBCT at different time points after immediate implant placement (**Figure 5**).

## Discussion

In this study, the overall 1-year implant survival rate was 97.86%, with 5 implants failing during the follow-up period. Stage-based analysis showed that survival rates were highest in stage II (100%) and gradually decreased in stage III (99.09%) and stage IV (93.65%). Although significant differences were observed, the survival rates for implants in stage III and stage IV were still relatively high. Based on a meta-analysis, the 1-year implant success rate in healthy individuals is reported to be 99.5% (Karl and Albrektsson, 2017). This suggests that even patients with stage II or III periodontitis can achieve favorable implant survival rates. However, stage IV periodontitis may require more careful consideration due to the potential impact on implant success. Similarly, survival rates were 100% in both grade A and B, but significantly lower in grade C (91.5%). The Log-Rank test confirmed that grade C had significantly lower survival rates compared to grades A and B, further emphasizing the importance of periodontal grade in predicting implant success. These findings underscore the need for careful patient selection based on the severity and grade of periodontitis to optimize the outcomes of immediate implant placement. Early research has demonstrated that periodontitis increases the risk of inflammation around implants, and that the microorganisms found around implants are similar to those found in periodontitis (Meffert, 1996). In the conventional delayed implant method, periodontitis is first treated systemically and implant surgery is then performed within six months, as this approach has been believed to increase the long-term success of the implant. However, patients with periodontitis have not been considered candidates for immediate implant placement due to concerns that microbes may interfere with the healing process, and that periapical and periodontal lesions may be contraindications for immediate extraction (Lindeboom et al., 2006; Chang et al., 2009).

Nevertheless, Crespi’s research has demonstrated that the inflammatory granulation tissue in the alveolar socket promotes bone healing, which is beneficial for osseointegration and has no adverse effects on the implanted implant (Crespi et al., 2017a; Crespi et al., 2017b). Therefore, the infected alveolar socket is not an absolute contraindication for immediate implant placement. In a prospective study, Crespi demonstrated that periodontitis does not increase the incidence of biological complications around the implant and that it also facilitates implant integration with the surrounding bone (Crespi et al., 2017b). Recent clinical studies have found that satisfactory clinical results can be achieved with immediate implantation in the presence of periodontal infection in the alveolar socket. Bone can form around the granuloma tissue after immediate implant placement in patients with periodontitis (Evian et al., 2004; Steiner et al., 2008). Furthermore, the fresh extraction socket provides an optimal implant bed, and the clinical treatment time is significantly shorter than that of delayed implant placement. Therefore, some researchers believe that immediate implant placement is a reasonable treatment option for patients with periodontitis. Existing literature also reports that several inflammatory factors stimulate bone regeneration after tooth extraction in periodontitis, which provides an optimistic prospect for immediate implant placement (Waasdorp et al., 2010; McCracken et al., 2012; Chrcanovic et al., 2015). Ashish’s research reveals a 95.4% success rate for 110 immediate implants in 60 periodontitis patients, utilizing laser decontamination, hardened bone grafts, and non-submerged healing, proving effective even in previously infected areas (Kakar et al., 2020).

To preserve the integrity of the alveolar ridge and facilitate optimal osseointegration, we utilized Bio-Oss bone graft (Geistlich, Switzerland) and Bio-Gide collagen membrane during immediate implant placement. These materials are essential for preventing bone resorption and ensuring adequate bone volume, particularly in sites with compromised bone architecture due to periodontitis. Their use is supported by previous studies, which have demonstrated that hard tissue augmentation can significantly improve outcomes in immediate implant procedures by promoting bone regeneration and maintaining the stability of the implant site.

Additionally, it was found that patients with periodontitis without systemic treatment had better clinical results compared to those who received systemic treatment. According to the standards developed by Alberksson and Zarb in 1986, the retention rate was good, and the bone absorption at the implant edge was no more than 2 mm (Albrektsson et al., 1986).

With the development of implant materials, immediate implant placement has become a satisfying surgery (Arghami et al., 2021). In a 20-year prospective study by Andrea, it was proposed that the implantation of tissue-level dental implants after comprehensive periodontal treatment and supplemented with supportive periodontal care (SPC) can lead to favorable long-term outcomes. However, it was observed that patients with a history of periodontitis who do not adhere to SPC have a higher risk of biological complications and implant loss (Roccuzzo et al., 2022). Therefore, when conditions permit, doctors should encourage patients to undergo periodontal treatment before implantation, especially those with severe periodontitis. The success rate of dental implants is also related to the patient’s oral hygiene habits (Cheung et al., 2021). Generally, periodontitis patients without systemic treatment or those with uncontrolled inflammation should have regular supportive periodontal therapy (SPT) after surgery (Iacono, 2000). Immediate implant placement was associated with high success rates in patients with or without periodontitis (Parvini et al., 2020).

Based on the findings of the present study, the stage of periodontitis, particularly Stage IV, was found to significantly influence the success rate of dental implants. This observation is consistent with several studies that have reported an increased risk of implant complications in patients with more advanced stages of periodontitis. Lindhe and Meyle (Lindhe et al., 2008) highlighted that higher-stage periodontitis is associated with greater peri-implant inflammation and a higher incidence of peri-implantitis, both of which can compromise implant survival. Similarly, Esposito et al. (2007) found that advanced periodontitis (Stage IV) significantly increases the likelihood of implant failure, primarily due to compromised bone quality and quantity, as well as an increased microbial load at the implant site.

However, the present study also found no significant differences in implant success between Stage II and Stage III periodontitis patients, suggesting that periodontitis in earlier stages does not substantially affect implant survival when appropriate care is taken. These findings support the notion that with proper management of risk factors, such as thorough cleaning, antimicrobial therapy, and bone augmentation when needed, immediate implant placement can be a viable and successful treatment option for patients across different stages of periodontitis.

It is important to note that, although Stage IV periodontitis does pose a higher risk to implant success, careful management and adjunctive treatments, such as bone grafting and antimicrobial therapy, can help mitigate these risks. Thus, immediate implant placement should be considered in a broader context, where both the stage and grade of periodontitis, along with the individual patient’s health status and risk factors, are carefully evaluated.

The success of immediate implants is also related to the patient’s lifestyle.

In addition to the stage and grade of periodontitis, smoking and diabetes are crucial factors that significantly influence the success of dental implants. Smoking is widely recognized as a major risk factor for implant failure, as it negatively impacts both soft and hard tissue healing. The literature consistently shows that smokers experience delayed wound healing, an increased risk of infection, and greater bone loss around implants compared to non-smokers.

Similarly, diabetes—particularly when poorly controlled—is another significant risk factor for implant failure. It leads to impaired immune function, delayed wound healing, and altered bone metabolism, all of which contribute to a higher rate of complications following dental implant placement.

When considering the combined effects of smoking, diabetes, and periodontitis, it becomes evident that these factors synergistically increase the risk of implant failure. Therefore, careful patient selection, smoking cessation, and strict glycemic control are essential for achieving favorable outcomes in immediate implant placement, particularly in patients with Grade C periodontitis. By addressing these modifiable risk factors, clinicians can significantly enhance implant success rates in patients with compromised periodontal conditions.

The primary objective of this study was to evaluate the success rate of immediate implant placement in patients with different periodontal conditions and to explore its feasibility and clinical limitations in the absence of systematic periodontal treatment. Accordingly, we enrolled 95 patients with varying stages and grades of periodontitis. During the initial consultation, patients were advised to undergo systematic periodontal therapy before proceeding with implant placement. However, the majority of patients prioritized a shorter treatment duration and immediate functional restoration, opting for implant placement despite their existing periodontal status. Therefore, respecting patient preferences, this study was conducted to delineate the extent to which immediate implantation is viable across different stages and grades of periodontitis while maintaining a high success rate.

Although patients in this study did not receive comprehensive periodontal therapy prior to implant placement, all underwent basic postoperative periodontal management, including scaling and comprehensive oral hygiene education. Previous studies have demonstrated that effective postoperative maintenance, particularly supportive periodontal therapy (SPT), plays a critical role in preventing peri-implant disease and ensuring long-term implant survival. Our findings further confirm that with rigorous infection control, precise surgical techniques, and adequate postoperative care, immediate implant placement can achieve a high success rate even in the presence of periodontitis.

Nevertheless, the optimal treatment protocol remains systematic periodontal therapy followed by delayed implant placement. However, in clinical practice, many periodontitis patients present at their first consultation with severe periodontal destruction, significantly compromising masticatory function and social confidence. In such cases, their primary concern is to restore function as quickly as possible, making immediate implant placement an attractive treatment option. This phenomenon is particularly evident in developing countries such as China, where limited oral health education and poor patient compliance often result in patients seeking treatment only at an advanced disease stage. Therefore, the findings of this study provide valuable clinical insights for practitioners managing similar patient populations.

Follow-up evaluations revealed high patient satisfaction with the outcomes of immediate implantation. Even in cases where implant failure occurred, subsequent reimplantation achieved favorable results. This further supports the notion that even in periodontitis patients, immediate implant placement can yield satisfactory clinical outcomes when combined with aggressive infection control, optimized surgical techniques, and comprehensive postoperative management. Consequently, for periodontitis patients requiring expedited functional rehabilitation, immediate implant placement remains a viable and effective treatment option.

This study has several limitations:

1. Limitations of a retrospective study: As a retrospective study based on existing case records, data collection was inherently constrained by missing information and potential selection bias.
2. Short follow-up duration: The study followed patients for one year, primarily assessing the short-term clinical outcomes of immediate implant placement in different periodontal conditions. However, the long-term survival of implants and their impact on the incidence of peri-implant diseases require extended follow-up studies for further validation. Future research should incorporate longitudinal studies to comprehensively evaluate the long-term outcomes of immediate implant placement in periodontitis patients.
3. Limitations of periodontal classification methodology: This study utilized the 2018 classification system from the American Academy of Periodontology (AAP) and the European Federation of Periodontology (EFP) to categorize the periodontal status of extracted teeth. The Stage (disease severity) was determined based on marginal bone loss (MBL, % of root length) assessed via CBCT imaging and periodontal probing before extraction, while the Grade (progression rate) was estimated using annual bone loss relative to patient age, smoking status, and diabetes history. Although this classification effectively reflects the severity and progression of periodontitis, the lack of long-term imaging data (e.g., a continuous record of periodontal health status and alveolar bone resorption) limits the accuracy of bone loss rate estimation, which was instead inferred from age rather than directly measured. Additionally, while standardized CBCT protocols were employed to evaluate MBL, inter-individual variability in imaging measurements may have influenced Stage assignment. Future studies should incorporate longitudinal CBCT imaging to further validate the impact of different Stages and Grades on the long-term outcomes of immediate implantation (Tonetti et al., 2018b).
4. Incomplete systemic health and lifestyle data: This study did not systematically collect detailed medical histories (e.g., hypertension, cardiovascular diseases, neurological disorders) or lifestyle factors (e.g., sleep habits, alcohol consumption, family history of periodontitis, psychological stress), which may influence periodontal disease progression and implant survival. Additionally, while the Grade classification considered bone loss rate, smoking, and diabetes, it did not account for other potential contributing factors such as genetic predisposition or microbiome variations. Future research should incorporate a broader dataset and apply multivariate regression models to refine the assessment of risk factors influencing implant outcomes.
5. Future research directions: To further investigate the long-term clinical performance of immediate implant placement in periodontitis patients, a prospective longitudinal study with extended follow-up is planned. This study will utilize Kaplan-Meier survival analysis and Cox proportional hazards modeling to assess the impact of Stage, Grade, lifestyle factors, systemic health conditions, and racial/ethnic differences on implant survival. These findings will help refine clinical decision-making for immediate implant placement in periodontitis patients.

## Conclusion

This study evaluated the success rate of immediate implant placement in patients with different periodontal conditions and examined the impact of Stage (severity of periodontitis) and Grade (disease progression rate) on implant survival and peri-implant tissue health (mSBI, mPLI, PPD, and MBL). The results demonstrated that under strict infection control, precise surgical techniques, and proper postoperative management, immediate implant placement achieved a high short-term success rate across various stages of periodontitis. However, patients with Stage IV periodontitis exhibited significantly greater marginal bone loss (MBL) compared to those with Stage II and Stage III (p < 0.05), along with trends in peri-implant probing depth (PPD), indicating that disease severity affects peri-implant hard tissue conditions.

Among these factors, Grade had a stronger influence on implant survival, with Grade C patients showing a significantly lower survival rate than those in Grade A and B (p < 0.05). This suggests that factors such as smoking and diabetes may further increase the risk of implant failure, underscoring the need for comprehensive risk assessment in periodontitis patients undergoing immediate implantation.

Although this study supports the feasibility of immediate implant placement in periodontitis patients, a more stringent patient selection process, preoperative intervention, and postoperative management are required for high-risk individuals (e.g., Stage IV, Grade C) to optimize long-term implant survival. Future studies should incorporate prospective long-term follow-up and apply Kaplan-Meier survival analysis and Cox proportional hazards modeling to further explore the impact of periodontal status, lifestyle factors, and systemic health conditions on implant longevity, ultimately refining clinical strategies for immediate implant placement in periodontitis patients, particularly those at high risk, where disease control and long-term maintenance remain key challenges.

## Data Availability

The original contributions presented in the study are included in the article/supplementary material. Further inquiries can be directed to the corresponding author.
